# Comparative chloroplast genomics provides insights into the genealogical relationships of endangered *Tetraena mongolica* and the chloroplast genome evolution of related *Zygophyllaceae* species

**DOI:** 10.3389/fgene.2022.1026919

**Published:** 2022-12-08

**Authors:** Yanci Yang, Yun Jia, Yanling Zhao, Yonglong Wang, Tao Zhou

**Affiliations:** ^1^ School of Biological Science and Technology, Baotou Teachers’ College, Baotou, China; ^2^ Xi’an Botanical Garden of Shaanxi Province, Institute of Botany of Shaanxi Province, Xi’an, Shaanxi, China; ^3^ School of Pharmacy, Xi’an Jiaotong University, Xi’an, Shaanxi, China

**Keywords:** chloroplast evolution, phylogenetic inference, *Tetraena mongolica*, *ndh* gene family, *Zygophyllaceae*

## Abstract

A comprehensive understanding of genetic background for rare species will provide an important theoretical basis for the future species management, monitoring and conservation. *Tetraena mongolica* is restrictedly distributed in the western Ordos plateau of China and has been listed as a national protected plant. We generated 13 chloroplast (cp) genomes of *T. mongolica* (size range of 106,062–106,230 bp) and conducted a series of comparative analyses of six *Zygophyllaceae* cp genomes. *T. mongolica* cp genome exhibited a quadripartite structure with drastically reduced inverted repeats (IRs, 4,315 bp) and undergone the loss of a suit of *ndh* genes and a copy of rRNAs. Furthermore, all the *T. mongolica* populations were divided into two genetic groups based on complete cp phylogenomics. In addition, notably variable genome size, gene order and structural changes had been observed among the six *Zygophyllaceae* cp genomes. Overall, our findings provide insights into the cp genome evolution mode and intraspecific relationships of *T. mongolica*, and provide a molecular basis for scientific conservation of this endangered plant.

## Introduction

Dramatic global climate change and human activities have triggered the loss of biodiversity and the sixth mass extinction (Barnosky et al., 2011). It is noteworthy that rare species—that is, represented by only a few individuals or restricted to limited areas—act as the cornerstones of the community they support, however, they would be vulnerable to extinction when against the climate change and anthropogenic pressures (Bracken and Low, 2012; Mouillot et al., 2013). Thus, a comprehensive understanding of genetic background for species, especially for rare species, is essential, which will provide an important theoretical basis for the future species management, monitoring and conservation.

*Tetraena mongolica* Maxim, a super-xerophilous shrub, is a monotypic genus of *Zygophyllaceae* (Zhao, 1997). *T. mongolica* is considered as a Tertiary relic species with extant populations are restrictedly distributed in the western Ordos plateau of China (Wang, 2005). In addition, intensifying anthropogenic activities, such as urbanization, grazing, mining and woodcutting, all contributed to the significantly population declined and habitat fragmentation of *T. mongolica* in recent decades (Wang, 2005). In China, *T. mongolica* has been listed as a national protected species for its higher extinction risk (Fu, 1992).

Previous molecular studies based on a limited number of markers have provided insights into the genetic diversity, genetic structure and phylogenetic relationships of *T. mongolica* (Ge et al., 2011; Zhi et al., 2018). However, both of the studies suffered from a lack of resolution for intraspecific relationships, which may be largely related to the insufficient phylogenetic signals. Furthermore, based on numerous SNPs from Genotyping-by-Sequencing, Cheng et al. (2020) revealed that *T. mongolica* was divided into two groups but with lower support values. To obtain more comprehensively understanding of this endangered species, we will explore the related evolutionary questions with complete chloroplast (cp) genome, which can provide more phylogenetic signals compared with a limited number of markers and reflect the evolutionary history of a single parental lineage.

Sequencing technical feasibility has made cp genome a workhorse of plant phylogeny and genome evolution (e.g., Song et al., 2017; Sullivan et al., 2017; Fu et al., 2019; Zhai et al., 2019; Cauz-Santos et al., 2020; Guo et al., 2021a; Yang et al., 2021). Given that the advantages of cp genomes, such as moderate genomic size, generally recombination free and uniparental inheritance (Birky et al., 1983; Wolfe and Randle, 2004), they act as a cornerstone in plant phylogenetic inferences. Most frequently, cp phylogenomics has been rendered fully resolved phylogenetic relationships, even in enigmatic plant lineages (Ma et al., 2014; Zhang et al., 2017; Fu et al., 2019; Yang et al., 2021). Most cp genomes of land plants show a simple mode of evolution, which is mainly characterized by highly conserved genome structure and organization. It has been well documented that most cp genomes have a typical quadripartite structure, with two copies of inverted repeat (IR) regions separating the small and large single copy (SSC and LSC, respectively) regions (Jansen et al., 2005; Jansen and Ruhlman, 2012). Additionally, most cp genomes encode around 101–118 distinct genes distributed over 120–170 kb (Bock, 2007; Jansen and Ruhlman, 2012; Ruhlman and Jansen, 2014). While exceptions to the simple mode of cp evolution have been observed, exemplifying genomic upheaval resulting from gene loss (e.g., Lauraceae, Song et al., 2017), IR expansion/contraction or loss (e.g., *Paphiopedilum*, Guo et al., 2021a; Geraniaceae, Weng et al., 2014) and rearrangements (e.g., *Passiflora*, Cauz-Santos et al., 2020). Moreover, high frequency of hybridization and biparental inheritance give rise to recombination of cp genomes (e.g., *Picea*, Sullivan et al., 2017).

In this study, we generated 15 cp genomes representing three species of *Zygophyllaceae* comprising 13 accessions of *T. mongolica* and one each of *Zygophyllum mucronatum*, *Zygophyllum xanthoxylon*. We intend to 1) explore the cp genome evolution mode of *T. mongolica* by comparing with all genera for which reliable cp genomes have been published in *Zygophyllaceae*; and 2) estimate the intraspecific relationships of *T. mongolica* with different data partitions.

## Materials and methods

### Taxon sampling and plant material

Leaves were collected of *T. mongolica*, *Z. mucronatum*, and *Z. xanthoxylon*. Detailed information of sampling is provided in Table 1. The geographical coordinates of 13 locations were collected from field investigations, which covered most of the known area of *T. mongolica*. Currently, the best-supported taxonomy of *Z. mucronatum* and *Z. xanthoxylon* is that they belong to genus *Zygophyllum* (e.g., Beier et al., 2003; Wu et al., 2018; Zhang et al., 2021), whereas *Z. xanthoxylon* has been categorized into genus *Sarcozygium* by Bunge (1843) and Liou (1998). Thus, we collected *Z. mucronatum* and *Z. xanthoxylon* for comparative analyses. Totally, we generated 15 new cp genomes, including 13 *T. mongolica* cp genomes, one *Z. mucronatum* cp genome and one *Z. xanthoxylon* cp genome. Additionally, three previously published cp genomes of *Tribulus terrestris* (Yan et al., 2019), *Larrea tridentata* and *Guaiacum angustifolium* (Gonçalves et al., 2019) are retrieved in this study, representing all genera for which reliable cp genomes have been published in *Zygophyllaceae* (Table 1).

**TABLE 1:** Information of samples in this study.

Species	Populations	Collection locality	Latitude (N°)	Longitude (E°)	Elevation (m)	GenBank number
*Tetraena mongolica*	WD01	Wudaqu, Wuhai, Inner Mongolia	39.47	106.66	1,195	ON408217
	QEG09	Hainanqu, Wuhai, Inner Mongolia	39.30	106.81	1,094	ON408220
	QPJ11	Etuokeqi, Ordos, Inner Mongolia	39.41	106.98	1,259	ON408221
	HNHXQ15	Hainanqu, Wuhai, Inner Mongolia	39.54	106.89	1,285	ON408222
	HNHCQ39	Hainanqu, Wuhai, Inner Mongolia	39.45	106.77	1,091	ON408229
	SC04	Haibowanqu, Wuhai, Inner Mongolia	39.66	106.85	1,165	ON408218
	MEG07	Haibowanqu, Wuhai, Inner Mongolia	39.68	106.88	1,239	ON408219
	QLG21	Haibowanqu, Wuhai, Inner Mongolia	39.88	106.93	1,311	ON408223
	MX23	Haibowanqu, Wuhai, Inner Mongolia	39.85	106.87	1,231	ON408224
	QPC25	Etuokeqi, Ordos, Inner Mongolia	39.90	106.75	1,089	ON408225
	TST30	Etuokeqi, Ordos, Inner Mongolia	40.17	106.92	1,062	ON408226
	SGK32	Hangjingqi, Ordos, Inner Mongolia	40.25	107.12	1,197	ON408227
	WJRGC35	Hangjingqi, Ordos, Inner Mongolia	40.22	107.34	1,214	ON408228
*Tribulus terrestris*	—	—	—	—	—	MN164624
*Larrea tridentata*	—	—	—	—	—	MK726018
*Guaiacum angustifolium*	—	—	—	—	—	MK726011
*Zygophyllum mucronatum*	—	Shandan, Zhangye, Gansu	38.43	101.14	2,289	ON408230
*Zygophyllum xanthoxylon*	—	Hainanqu, Wuhai, Inner Mongolia	39.54	106.89	1,285	ON408231

### DNA extraction, sequencing, assembly and annotation

Total genomic DNA was extracted for the 15 accessions from silica-dried leaf material following the modified CTAB method (Doyle, 1987). Sequencing was completed on an Illumina Hiseq platform, yielding at least 2 GB clean data for each accession. All of the above work were conducted by Biomarker Technologies Inc (Beijing, China). Clean reads were assembled using MIRA 4.0.2 (Chevreux et al., 2004) and MITObim v1.7 (Hahn et al., 2013) with default settings. In this process, the published cp genome of *T. mongolica* (MK331720), *Z. xanthoxylon* (MZ427318) and *Zygophyllum fabago* (MK341052) were used as reference genome in cp genome assembly of *T. mongolica*, *Z. xanthoxylon* and *Z. mucronatum*, respectively. Annotation of the cp genomes was performed in GENEIOUS v.11 (Biomatters Ltd., Auckland, New Zealand) coupled with manual adjustments.

### Sequence divergence, genome rearrangement and IR/SC boundary analysis

Genetic diversity of *T. mongolica* was assessed. Sequences were aligned by MAFFT with the default settings (Katoh and Standley, 2013). Variable sites, informative sites, nucleotide diversity (Pi) and indels in the aligned result were detected by DnaSP v 5.0 (Librado and Rozas, 2009). Furthermore, Progressive Mauve v 2.4.0 (Darling et al., 2004) was used to identify the possible rearrangements among the six *Zygophyllaceae* cp genomes. The six *Zygophyllaceae* cp genome alignment was visualized using mVISTA with the sequence of *T. terrestris* as the reference (Frazer et al., 2004). In addition, to detect possible expansion or contraction in junction regions, the IR/SC boundaries were analyzed for both *T. mongolica* cp genomes and *Zygophyllaceae* cp genomes.

### Codon usage bias analysis

To avoid sampling bias, protein-coding regions with the length greater than 300 bp were extracted for the analysis (Wright, 1990). Finally, the number of selected protein-coding genes of *T. terrestris*, *L. tridentata*, *G. angustifolium*, *T. mongolica*, *Z. mucronatum*, and *Z. xanthoxylon* were 58, 57, 56, 41, 41, and 41, respectively. Relative synonymous codon usage (RSCU) is the ratio of the observed frequency of a codon to the expected frequency and is a good indicator of codon usage bias (Sharp and Li, 1986). RSCU values of the six *Zygophyllaceae* cp genomes were calculated by MEGA v 5.0 (Tamura et al., 2011). The heatmap from all RSCU values was carried out using Morpheus (https://software.broadinstitute.org/morpheus).

### Repeat elements and SSRs analysis

Dispersed repeats, tandem repeats and simple sequence repeats (SSRs) within six *Zygophyllaceae* cp genomes were analyzed, respectively. Firstly, REPuter (Kurtz et al., 2001) was used to identify dispersed repeats, including forward, reverse, complement and palindromic repeats. We focused on the dispersed repeats having a minimal size of 30 bp and 90% or greater similarity between the two repeat copies. The maximum distance between palindromic repeats is 3 kb. Subsequently, tandem repeats (>10 bp in length) were detected using online program Tandem Repeats Finder (Benson, 1999) with default parameters. The minimum alignment score and maximum period size were set as 50 and 500, respectively. All found repeats were manually verified and the redundant results were removed. Finally, SSRs were detected by msatcommander (Faircloth, 2008). The minimum repeat unit were 10, 5, 4, 3, 3, and 3 for mono-, di-, tri-, tetra-, penta-, and hexanucleotides, respectively.

### Phylogenetic inference and network analysis

Phylogenetic analyses of different datasets were conducted using Maximum likelihood (ML) and Bayesian inference (BI) methods, which were conducted using RAxML v7.2.8 (Stamatakis, 2006) and MrBayes v3.1.2 (Ronquist and Huelsenbeck, 2003), respectively. To estimate the phylogenetic relationships of *T. mongolica*, the following datasets: 1) complete cp genome sequences (CCS); 2) noncoding region sequences (NCS); and 3) protein-coding region sequences (PCS) were analyzed. In these inferences, *L. tridentata* (MK726018) and *G. angustifolium* (MK726011) were treated as outgroups. For *Zygophyllaceae*, we used *Krameria bicolor* (MK726015) and *Krameria lanceolata* (MK726016) as outgroups. Datasets:1) complete cp genome sequences (CCS); and 2) SSC + single IR sequences (SIS) were conducted for phylogenetic analysis. Sequence alignment was performed using MAFFT (Katoh and Standley, 2013) with the default parameters set. The ML tree was inferred with GTR + G model and 1,000 bootstrap replicates. The best-fitting model for BI analyses was determined using Modeltest 3.7 (Posada and Crandall, 1998) based on the Akaike information criterion. Two independent Markov chain Monte Carlo runs were performed for one million cycles with sampling every 100 generations, and the first 25% of the trees were discarded as burn-in.

A TCS network of intraspecific relationships of *T. mongolica* was performed by PopArt v 1.7 (Leigh and Bryant, 2015) using the 13 cp genome haplotypes identified by DnaSP v 5.0 (Librado and Rozas, 2009).

### Divergence time of *T. mongolica*

The divergence times among *T. mongolica* lineages were estimated using BEAST v1.8.0 (Drummond and Rambaut, 2007). Dating analysis were conducted under GTR substitution model and an uncorrelated lognormal relaxed clock. A Yule process was chosen to model speciation. Thirty million generations were run of the two independent Markov chain Monte Carlo, with sampling every 3000th generation. Finally, the stationarity of the tree was checked using Tracer v1.5 by assessing the effective sample size (ESS) values (>200). Final trees were edited using FigTree v1.4.3 (http://beast.community/figtree). Considering the ambiguity of *Zygophyllaceae* fossil record (Bellstedt et al., 2012), we estimated the divergence time twice. Firstly, the stem age of *T. mongolica* was set at 9.38 Ma and the split between *L. tridentata* and *G. angustifolium* was set at 17.22 Ma, as the results of Wu et al. (2015). Secondly, the split between *Zygophyllaceae* and *Krameriaceae* was 92.1 Ma following the results of Li et al. (2019). Hence, *K. bicolor* and *K. lanceolata* were retrieved as the outgroups.

## Results

### Characteristics of *T. mongolica* cp genomes

To assess intraspecific variation within *T. mongolica*, we sampled 13 populations of *T. mongolica* covered most of the known area of this endangered plant. A total of 13 complete cp genomes were newly generated by using the next generation sequencing approach (Table 1). *T. mongolica* cp genomes were highly conserved and all of them displayed the typical quadripartite structure, including LSC, SSC, and two copies of IR regions. The complete cp genome size ranged from 106,062 bp (WJRGC35) to 106,230 bp (MX23) (Table 2). In the LSC region, the length varied from 80,273 bp (WJRGC35) to 80,351 bp (QPC25), in the SSC region from 17,159 bp (WJRGC35) to 17,268 bp (HNHCQ39). Notably, the length of IR was stable among different accessions (4,315 bp). The IR/SC boundaries of these cp genomes were stable, with the only exception that the distance from the *trnL* (*UAG*) end to the junction of IRa/SSC boundary of HNHCQ39 and the other accessions were 555 bp and 553 bp, respectively (Supplementary Figure S1). The GC content of 13 cp genomes ranged from 33.60% to 33.63%, and averaged 33.61% (Table 2).

**TABLE 2:** Summary of the 13 *T. mongolica* chloroplast genomes.

Accessions	LSC (bp)	SSC (bp)	IRa (bp)	IRb (bp)	Size (bp)	GC content (%)	Protein-coding genes	tRNA genes	rRNA genes	Total genes
WD01	80,340	17,200	4,315	4,315	106,170	33.61	67	33	4	104
QEG09	80,326	17,200	4,315	4,315	106,156	33.62				
QPJ11	80,334	17,199	4,315	4,315	106,163	33.60				
HNHXQ15	80,349	17,200	4,315	4,315	106,179	33.61				
HNHCQ39	80,280	17,268	4,315	4,315	106,178	33.62				
SC04	80,311	17,243	4,315	4,315	106,184	33.61				
MEG07	80,302	17,244	4,315	4,315	106,176	33.61				
QLG21	80,340	17,255	4,315	4,315	106,225	33.60				
MX23	80,337	17,244	4,315	4,315	106,230	33.60				
QPC25	80,351	17,200	4,315	4,315	106,181	33.61				
TST30	80,284	17,245	4,315	4,315	106,159	33.61				
SGK32	80,284	17,263	4,315	4,315	106,177	33.61				
WJRGC35	80,273	17,159	4,315	4,315	106,062	33.63				

*T. mongolica* composed of 104 genes, including 67 protein-coding genes, 33 tRNA genes and four rRNA genes (Table 2; Supplementary Table S1). In addition, a total of 77 genes were located in LSC, including 56 protein-coding genes and 21 tRNAs. SSC harbored five protein-coding genes, six tRNAs and four rRNAs. The remaining genes were located in IRs.

The 13 complete cp genomes had an aligned length of 106,471 bp, while there were only 128 variable sites and 41 parsimony-informative sites. Nucleotide diversity (Pi) across the complete cp genomes of total populations was relatively low (Pi = 0.00032). In contrast, non-coding regions showed the highest sequence diversity (Pi = 0.00054) and protein-coding genes exhibited extremely low sequence diversity (Pi = 0.00012) (Table 3).

**TABLE 3:** Genetic diversity of *T. mongolica*.

	Complete cp genomes	Protein-coding genes	Non-coding regions
Number of sites	106,471	46,764	52,798
Number of variable/informative sites	128/41	21/7	104/35
Nucleotide diversity (Pi)	0.00032	0.00012	0.00054
Number of indels	527	0	533

### Characteristics of *Zygophyllaceae* cp genomes

To investigate the evolution mode of *T. mongolica* cp genome, a total of six species of *Zygophyllaceae* were analyzed, including *T. terrestris* (subfam. Tribuloideae), *L. tridentata* (subfam. Larreoideae), *G. angustifolium* (subfam. Larreoideae), *T. mongolica* (subfam. Zygophylloideae), *Z. mucronatum* (subfam. Zygophylloideae) and *Z. xanthoxylon* (subfam. Zygophylloideae), which covered all genera for which reliable cp genomes have been published in Zygophyllaceae. Due to the highly conserved cp genomes, we used WD01 to represent *T. mongolica* for the subsequent analyses.

The complete cp genome sequences of six Zygophyllaceae species were 105,251 bp (*Z. mucronatum*) to 158,184 bp (*T. terrestris*) (Table 4). All of them exhibited a typical quadripartite structure, including a pair of IR regions (8,576–51,684 bp), one LSC region (79,912–88,878 bp) and one SSC region (13,767–17,622 bp). The GC contents of six cp genomes ranged from 33.61% (*T. mongolica*) to 35.81% (*T. terrestris*).

**TABLE 4:** Summary of the six Zygophyllaceae chloroplast genomes.

	*T. terrestris*	*L. tridentata*	*G. angustifolium*	*T. mongolica*	*Z. mucronatum*	*Z. xanthoxylon*
Subfamily	Tribuloideae	Larreoideae	Larreoideae	Zygophylloideae	Zygophylloideae	Zygophylloideae
Size (bp)	158,184	135,988	130,809	106,170	105,251	105,802
LSC (bp)	88,878	81,323	81,554	80,340	79,912	80,263
SSC (bp)	17,622	15,965	13,767	17,200	16,513	16,963
IRs (bp)	51,684	38,700	35,488	8,630	8,826	8,576
Overall GC content (%)	35.81	35.08	35.57	33.61	33.90	33.98
Total genes	130	129	129	104	104	104
Protein-coding genes	84	80	80	67	67	67
tRNA genes	37	37	37	33	33	33
rRNA genes	8	8	8	4	4	4
pseudogene(s)	1	4	4	0	0	0

Due to the high conservation of cp genomes, we used WD01 (GenBank number: ON408217) to represent *Tetraena mongolica* for comparative analyses and phylogenetic inference.

**FIGURE 1:**
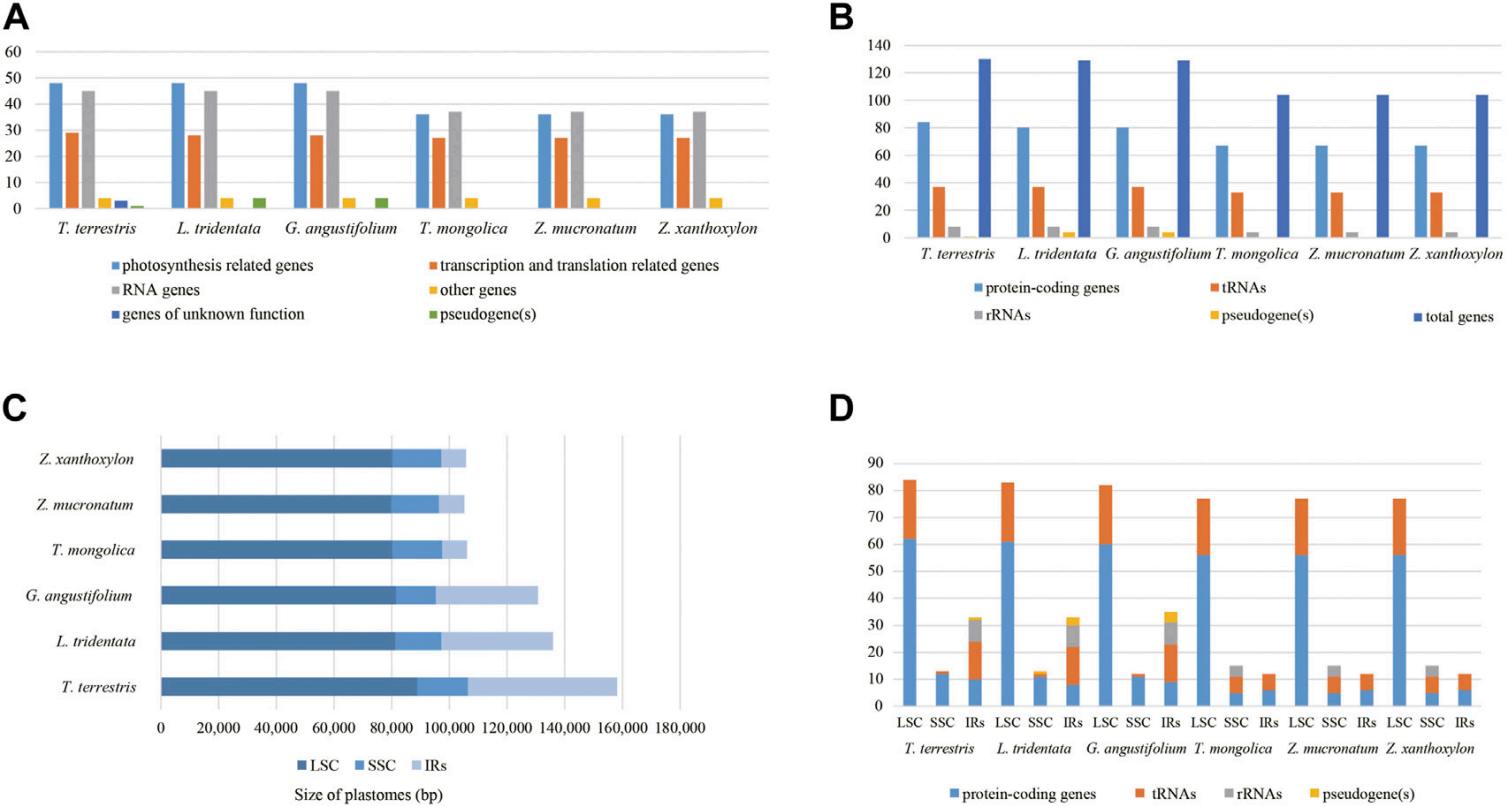
Characteristics of *Zygophyllaceae* chloroplast genomes. **(A)** Number of different functional categories of genes. **(B)** Number of genes. **(C)** Size of cp genomes. **(D)** Number of genes located in different regions.

**FIGURE 2:**
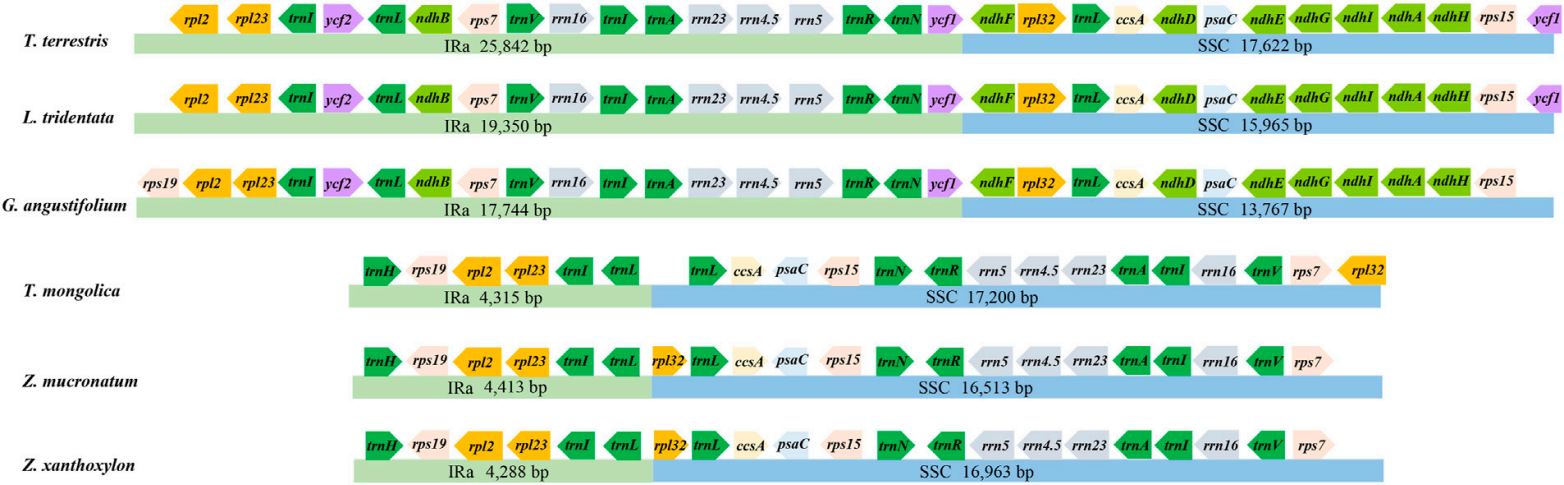
Genes in IRa and SSC of Zygophyllaceae chloroplast genomes.

**FIGURE 3:**
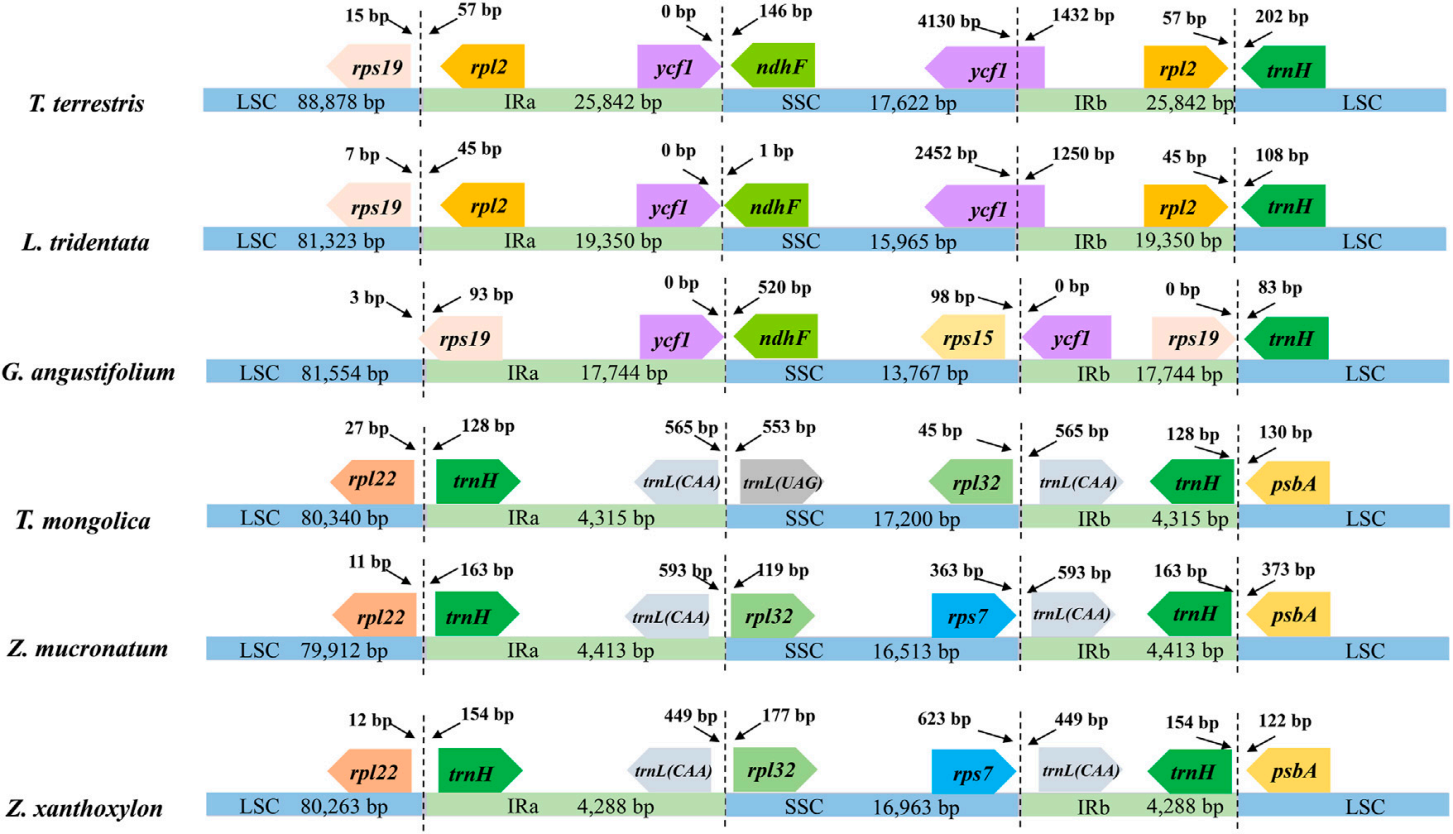
IR/SC boundaries of six Zygophyllaceae chloroplast genomes. Number above the gene features means the distance between the ends of genes and the borders sites. These features are not to scale.

The six cp genomes of *Zygophyllaceae* exhibited two patterns of gene order and content: the cp genomes of subfam. *Zygophylloideae* possessed basically identical genes, and the remaining three cp genomes shared similar coding genes. The specific details of gene loss were shown in the Supplementary Table S1. The LSC of the six cp genomes possessed remarkable resemblance in terms of gene order and content, encoding from 77 to 84 genes (Figure 1). While these cp genomes differed greatly in size, gene order and content of the remaining regions (Figure 2)—for instance, gene numbers varied from 12 (subfam. Zygophylloideae) to 35 (*G. angustifolium*) in IRs, which attributed to the gene loss (e.g., *ndh* gene family) and the transfer of genes from IRs to SSC region (e.g., rRNA genes). Although most IR boundary shifts are small (up to several hundred bp) among land plants (Zhu et al., 2016), significant IR/SC boundaries shifts occurred in Zygophyllaceae cp genomes, yielding strongly shrinking IRs in subfam. Zygophylloideae (Figure 3). Additionally, different from a broad range of land plants with conserved collinear gene order, and also from several lineages (e.g., Geraniaceae and Campanulaceae) with reconfigurable gene order (Cosner et al., 2004; Guisinger et al., 2011; Weng et al., 2014), the six Zygophyllaceae cp genomes had relatively few rearrangement events as indicated in the Mauve alignment (Supplementary Figure S2). As expected, there were much more highly divergent sequences in non-coding regions than in coding regions (Supplementary Figure S3).

### Codon usage analysis

**FIGURE 4:**
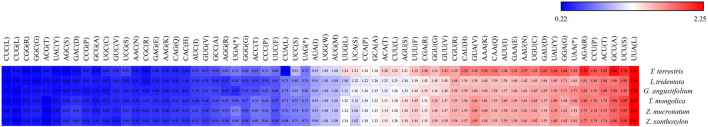
The RSCU values of protein-coding genes for six Zygophyllaceae species. Color key: the red values indicated higher values and the blue values indicated lower values.

To avoid sampling bias, protein-coding regions with the length greater than 300 bp were analyzed (Wright, 1990), resulting in a total of 13,936 (*Z. xanthoxylon*) to 24,733 (*T. terrestris*) codons. The codon usage and RSCU values of six *Zygophyllaceae* cp genomes were summarized in Supplementary Table S2 and the heatmap of RSCU values were shown in Figure 4. Although the number of codons varied dramatically for each amino acid among these species, the codon bias of each amino acid was largely consistent. For example, the amino acid Leu was encoded by six codons (UUA, UUG, CUU, CUC, CUA, and CUG), and the codon, UUA, was used on average 0.83 times less frequently in *T. terrestris* genes than in the remaining species genes, however, UUA was always the most frequently occurring codon (with the highest RSCU value) relative to the other codons encoding Leu in six species. Codons AUG and UGG showed no bias (RSCU = 1), as Methionine (Met) and Tryptophan (Trp) were encoded only by AUG and UGG, respectively. Concurrently, 30 codons were translation-preferred (RSCU>1) and characterized with a bias in favor of purine (A/U) at the third codon position (except UUG).

### Repeat sequences and simple sequence repeats

**FIGURE 5:**
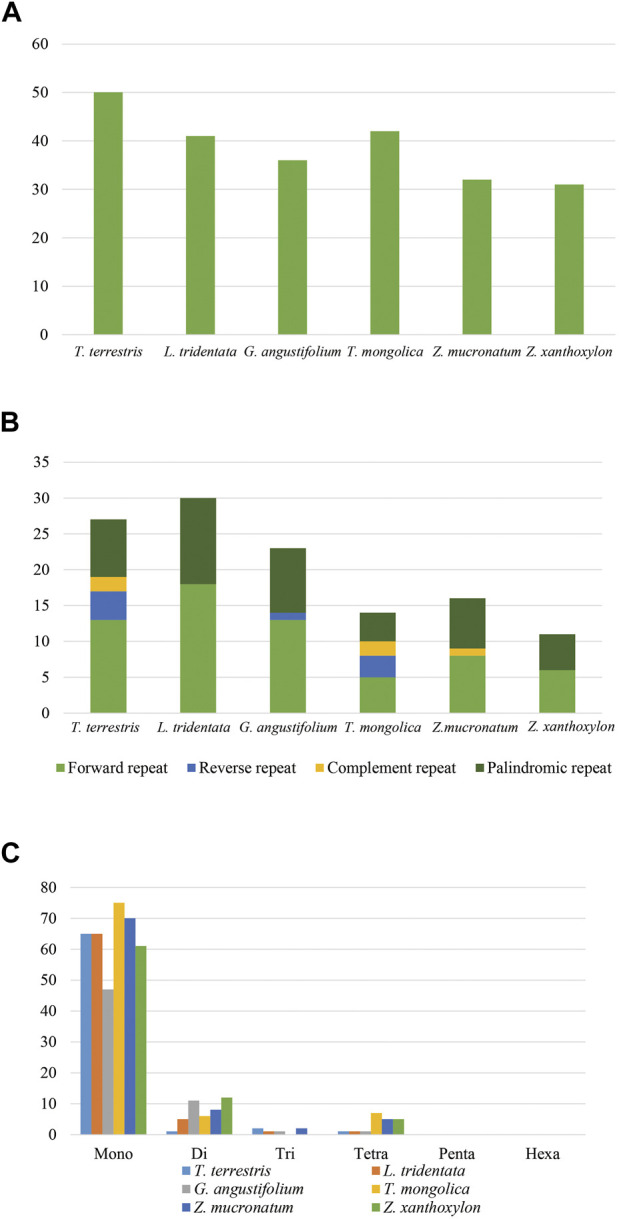
Numbers and types of repeat elements. **(A)** Tandem repeats. **(B)** Dispersed repeats. **(C)** SSRs.

A total of 226 tandem repeats, 121 dispersed repeats and 452 SSRs were identified in the six cp genomes (Figure 5) and the detailed information was shown in Supplementary Table S3. In regard to tandem repeats, *T. terrestris* contained the largest number of repeats (50) while *Z. xanthoxylon* had the fewest (25). We found that 96.9% of tandem repeats had the repeat motif less than five and only three of the 226 repeats had larger repeat unit (>100 bp). We detected 121 dispersed repeats, and the majority were forward repeats (52.07%), followed by palindromic repeats (37.2%). In contrast, reverse repeats and complement repeats were found with much lower frequency or even absent in some species. Moreover, there were 115 (95.0%) smaller dispersed repeats (<50 bp) while *Z. mucronatum* and *Z. xanthoxylon* had four and two larger dispersed repeats (>100 bp), respectively. Among the 452 SSRs, mononucleotide repeats accounted for the highest proportion (84.7%) and all consisted of A/T repeats. Penta- and hexanucleotide repeats were absent in all species.

### Phylogeny and TCS analysis

**FIGURE 6:**
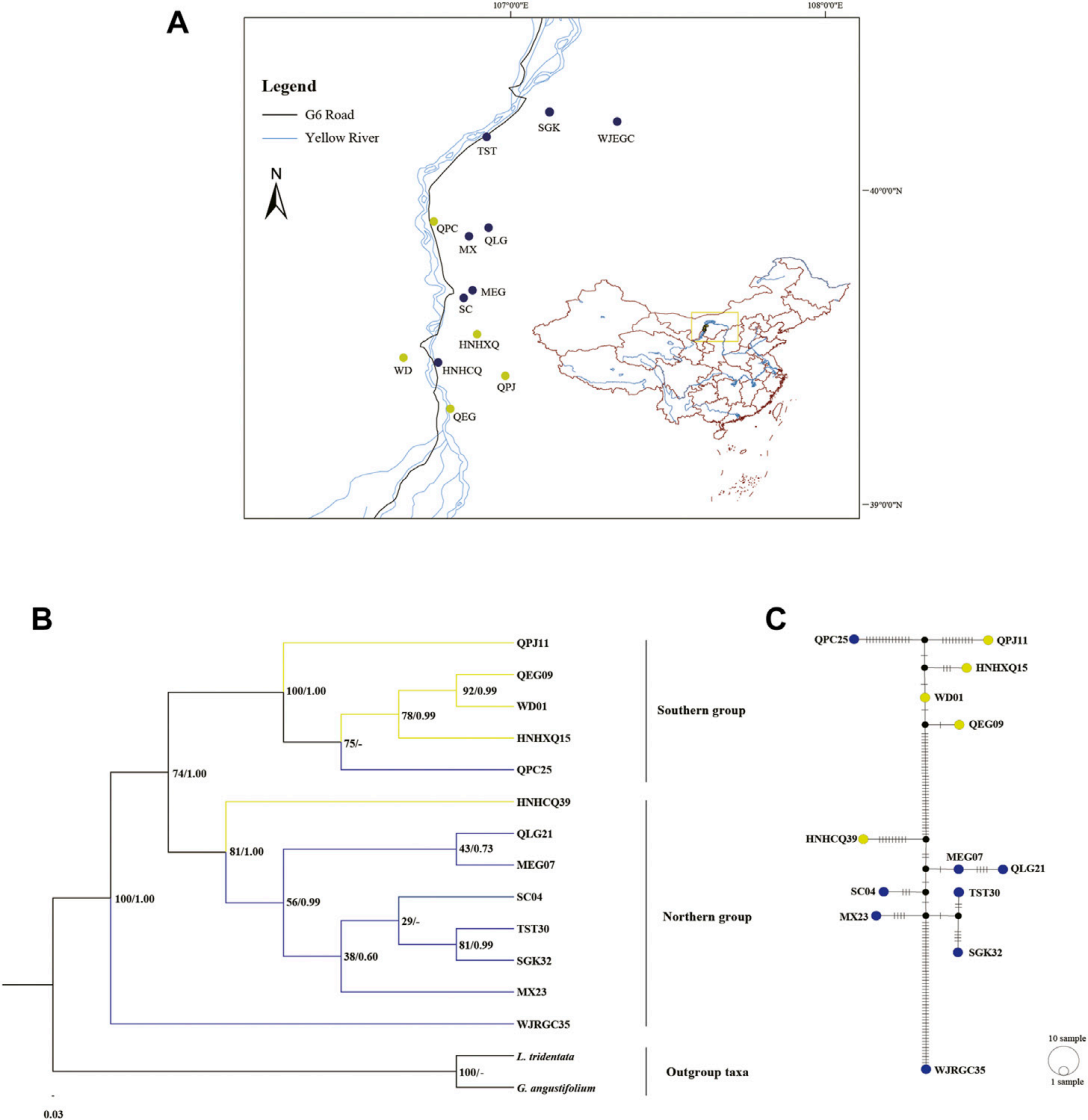
**(A)** Geographical distributions of sampled *T. mongolica* populations. **(B)** The ML tree of 13 *T. mongolica* inferred from complete chloroplast genomes. Numbers at nodes represented the bootstrap support values and posterior probability values. Dash (–) denoted this relationship was not supported by the BI analysis. **(C)** The TCS network of intraspecific relationships among the 13 cp haplotypes, the steps among haplotypes showed the degree of genetic variation.

Three datasets (CCS, NCS, and PCS) were used to reconstruct the intraspecific relationships of *T. mongolica* (Figure 6; Supplementary Figures S4,S5). The topologies estimated from ML and BI analyses of the CCS dataset and NCS dataset were broadly similar to each other and received moderate-to-high support. While the ML and BI topologies were generally incongruent inferred from PCS dataset with poor support for most nodes. Here, we reported the relationships from CCS dataset with higher support. Phylogenetic tree resolved two clades comprising: 1) one accession WJRGC35 and 2) the remaining accessions (Figure 6B). With the notable exception of QPC25 and HNHCQ39, accessions respectively belonging to northern populations and southern populations constituted the two subclades of the second clade. Notably, some nodes of the northern subclade received relatively low support values. Similarly, the TCS network result of the 13 cp haplotypes revealed a closer relationship of the most northern populations to southern populations than to WJRGC35 (Figure 6C). Overall, all *T. mongolica* populations were mainly divided into two groups: the northern group (WJRGC35 and northern populations) and the southern group (the remaining populations).

**FIGURE 7:**
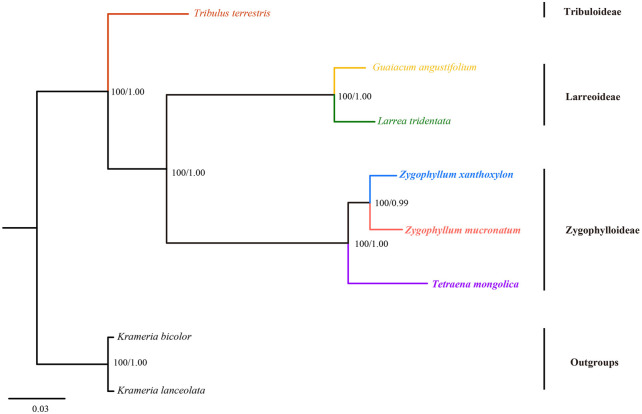
The ML tree of Zygophyllaceae inferred from complete cp genome dataset. Numbers at nodes represented the bootstrap support values and posterior probability values.

Due to the extensive variation of SSC and IRs, the phylogenetic analyses of *Zygophyllaceae* were performed based on CCS dataset (Figure 7) and SIS dataset (Supplementary Figure S6). Both ML and BI analyses based on the aforementioned two datasets recovered the same topologies with strong support. Three subfamilies were strongly supported and a closer relationship was exhibited between Larreoideae and *Zygophylloideae*. Furthermore, our present phylogenetic result showed the closest relationship between *Z. mucronatum* and *Z. xanthoxylon*.

### Divergence time estimation of *T. mongolica*

**FIGURE 8:**
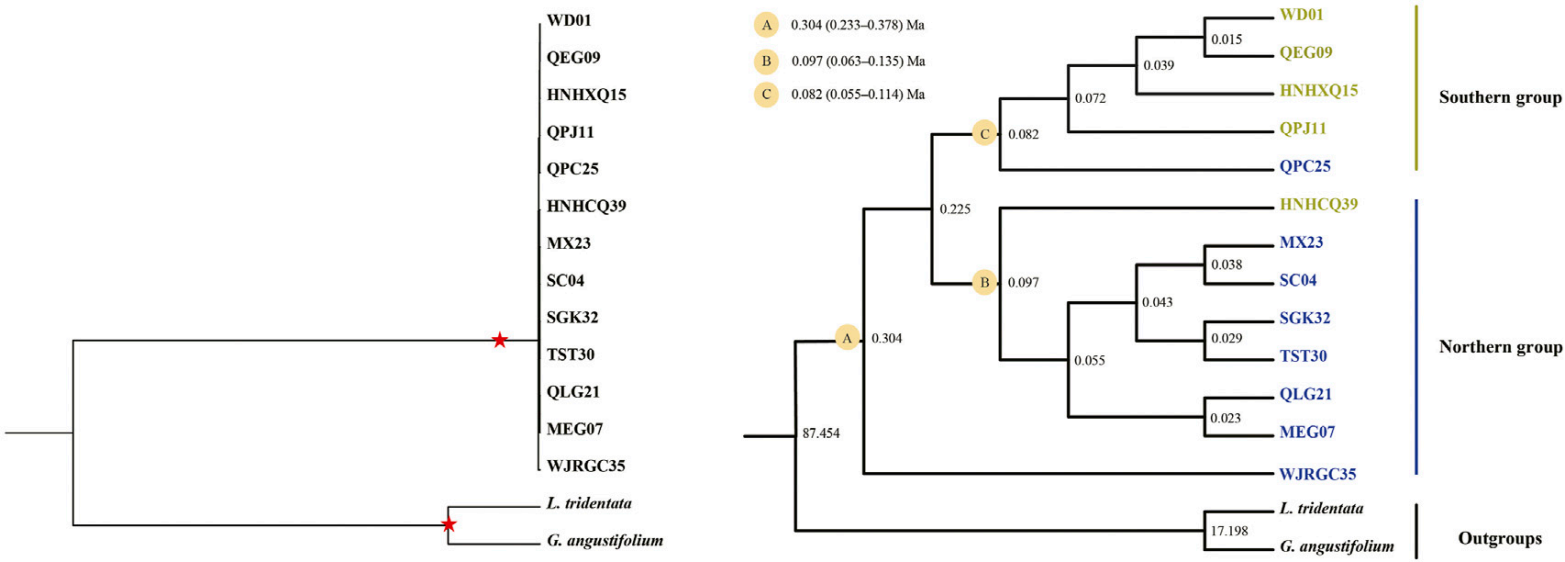
The BEAST-derived chronograms of *T. mongolica* with two calibration points. The divergence time estimates based on the complete cp genome sequences. Red pentastar represented the calibration points.

The BEAST-derived ages of *T. mongolica* with two calibration points (Figure 8) were largely agreed with those inferred by using one calibration point (Supplementary Figure S7). Thus, we focused on ages mainly from the former result. WJRGC35 diverged from the remainder at *ca.* 0.304 Ma (node A). The split between the mainly northern group and southern group was *ca*. 0.225 Ma. The onset of diversification of the mainly northern group and southern group occurred at 0.097 Ma (node B) and 0.082 Ma (node C), respectively.

## Discussion

### Unusual structure and gene loss events of the *T. mongolica* and *Zygophyllaceae* cp genomes

Despite the high conservation of cp genomes has been observed in most land plants, large-scale variations still have been detected, even within species. For example, extensive population divergences were observed in *Monotropa* and *Hypopitys*, suggesting that speciation may be occurring (Braukmann et al., 2017). For *T. mongolica*, 13 cp genomes possessed identical gene content and order, fairly close GC content, and extremely stable IR/SC boundaries. Additionally, extremely low sequence diversity was exhibited. Consequently, we found that cp genomes of *T. mongolica* were highly conserved, as observed in most land plants, such as *Leonurus Cardiaca* (Sun et al., 2021), *Cercidiphyllum japonicum* (Zhu et al., 2020).

Among the six cp genomes of *Zygophyllaceae*, genome length, organization and gene content of *T. mongolica* were more similar to the cp genome of *Z. xanthoxylon* and *Z. mucronatum* than to the remaining three species, exhibiting the drastically reduced IRs, the loss of *ndh* gene family and one copy of rRNAs.

Across the land plants, the significant reduction of cp genome size is mainly due to IR contraction/loss (e.g., IR-lacking clade (IRLC) legumes, some species of Geraniaceae; Duan et al., 2021; Guisinger et al., 2011) and gene loss (e.g., some parasitic plants and mycoheterotrophs; Braukmann et al., 2017; Graham et al., 2017). With the conspicuous exception of *T. terrestris* (∼158 kb), the remaining species in *Zygophyllaceae* had smaller cp genomes (*c.* 106–135 kb) compared with most land plants (an average size of 151 kb) (Guo et al., 2021b). Unlike most angiosperms retaining a 25 kb IR (Ruhlman and Jansen, 2014), variation in the size of the IR, which could range from 4,288 bp (*Z. xanthoxylon*) and up to 25,842 bp (*T. terrestris*), accounted for *Zygophyllaceae* cp genome size variation overall (105,251 bp to 158,184 bp). The phenomenon that IR has undergone expansion and contraction is nearly omnipresent while significant IR boundary shifts are relatively rare (Hansen et al., 2007; Yang et al., 2016; Zhu et al., 2016; Sinn et al., 2018; Wei et al., 2021). In all investigated six species except *T. terrestris*, the IR was thought to have undergone large-scale contraction relative to the most angiosperms (25 kb), contributing to their smaller IR size (4,288–19,350 bp). Overall GC content is typically in the range of 30–40% (average 37%) in most cp genomes of land plants (Bock, 2007; Civáň et al., 2014), and the average GC content (∼34.7%) of Zygophyllaceae cp genomes is relatively lower than in land plants (37%).

Moreover, the drastically reduced IR, rearrangement events, IR/SC boundary shifts all resulted in reconfiguration of gene content and order. The total gene numbers of the six *Zygophyllaceae* cp genomes differed greatly, from 104 in subfam. *Zygophylloideae*, to 130 in *T. terrestris*. Especially, gene numbers varied from 12 (subfam. Zygophylloideae) to 35 (*G. angustifolium*) located in IRs. Despite there were different numbers of protein-coding genes (67–84), the codon usage preference of each amino acid showed highly similar in these *Zygophyllaceae* cp genomes, even more broadly across land plants, as previous research results (e.g., Guisinger et al., 2011; Yang et al., 2018). We observed a similar bias in our analysis in comparison to many other land plants, the frequently used codons (RSCU value > 1) all but UUG ended in A/U (Kim and Lee, 2004). The presence of translation-preferred codons and A/U bias at the third codon position may be the result of both natural selection and mutation biases during the cp genome evolutionary process (Xu et al., 2011; Li et al., 2021).

Most remarkably, the loss of entire family of *ndh* genes (*ndhA*–*ndhK*) occurred in subfam. Zygophylloideae, which had also been detected and verified by PCR product sequencing (Wang et al., 2022). In most cp genomes of land plants, 11 genes (*ndhA*–*ndhK*) constitute *ndh* family and encode subunits of the NDH complex that is involved in photosystem I cyclic electron flow and chlororespiration (Burrows et al., 1998; Peltier and Cournac, 2002; Martín and Sabater, 2010; Peng et al., 2011). However, loss or pseudogenization of *ndh* gene family are well-known in a number of plant lineages. For example, these genes are commonly lost in non-photosynthetic plants and mycoheterotrophs (Graham et al., 2017; Sabater, 2021). In addition, it has been documented that *ndh* genes are lost in several fully photosynthetic plant lineages, such as Gnetales, several conifers (Martín and Sabater, 2010), *Carnegiea gigantea* (Sanderson et al., 2015), *Erodium* (Chris Blazier et al., 2011) and *Kingdonia* (Sun et al., 2017). For the loss of *ndh* genes in these fully photosynthetic plants, one possible reason is that the functional role of the protein products may be simply dispensable, especially for the taxa distributed in mild habitats (Martín and Sabater, 2010; Sabater, 2021). Alternatively, NDH-dependent pathway may be functionally replaced by another pathway (such as antimycin A-sensitive cyclic electron flow pathway), especially for the taxa under light, water and temperature stress (Sanderson et al., 2015). Notably, *Balanites aegyptiaca* (subfam. Tribuloideae, Zygophyllaceae), an arid and semi-arid plant, possesses all 11 *ndh* genes in cp genome, possibly indicating the different evolutionary history among the subfamilies of *Zygophyllaceae* (Al-Juhani et al., 2022). Specifically, considering the role of *ndh* genes in stress acclimation (Peng et al., 2011), angiosperms lacking *ndh* genes have a higher risk of extinction in facing variable evolutionary environments (Sabater, 2021) and may be occurring in endangered *Kingdonia uniflora* (Sun et al., 2017). When considering the loss of the *ndh* gene family, *T. mongolica* may have higher extinction risk in facing changing environments.

In most land plants, two copies of rRNAs (5S, 4.5S, 23S, and 16S rRNA) are often present in the IRa and IRb respectively. In subfam. *Zygophylloideae*, the highly unusual is that there is only one copy of rRNAs with SSC localization, which can be attributed mainly to the transfer of these genes from IRs to SSC rather than the loss of one copy of IR in several plant lineages, as observed for IRLC legumes. It has been detected in many land plants that genes transfer from the SC into the IRs and *vice versa* (Zhu et al., 2016), and the variation in the number of gene copy due to distribution changes is also present in some plants (e.g., Sinn et al., 2018; Henriquez et al., 2020).

### Repeat analyses

Larger and more complex repeat sequences may be correlated with the rearrangement of cp genomes (Timme et al., 2007; Weng et al., 2014). As observed in highly rearranged cp genomes, such as Geraniaceae (Weng et al., 2014) and *Passiflora* (Cauz-Santos et al., 2020), there are always existing a high frequency of larger repeats. In our study, there were few larger repeats (six of 121 dispersal repeats were longer than 100 bp), meanwhile, a low number of rearrangements were present in *Zygophyllaceae*. SSRs are ubiquitous throughout genomes and have been widely used as molecular markers (Kumar et al., 2015; Younis et al., 2020). Overall, SSRs present in our analyses could act as markers for genetic diversity and phylogenetic inference in *Zygophyllaceae*.

### Phylogenetic relationships in *T. mongolica* and *Zygophyllaceae*

Our phylogenetic result and TCS network result supported *T. mongolica* was mainly divided into two groups (northern group and southern group) with significantly improved support values, which is generally consistent with previous result inferred from cpDNA marker (Ge et al., 2011), rather than the result based on nuclear genomic data (Cheng et al., 2020). Although two groups were recognized by both cp genomes and nuclear genomic data, the populations located in the central of the distributional range were identified as the northern group and southern group based on cp genomes in our study and nuclear genomic data in Cheng et al. (2020), respectively. The incongruent phylogenetic topologies between nuclear and cp gene trees have been commonly observed, which may be triggered by incomplete lineage sorting (ILS), allopolyploidy, hybridization, introgression and so on (Duan et al., 2021). Interestingly, population WJRGC35 formed a clade that was sister to the remaining populations. The previous study suggested that the northernmost population (WJM population) may be the ancestral population of *T. mongolica* and spread southward in history (Cheng et al., 2020). In terms of geographical location, both SGK32 and WJRGC35 in our study belonged to the WJM population in the study of Cheng et al. (2020), while WJRGC35 was located in the central area of the WJM population. Accordingly, combined with our divergence time estimates, we supported that the ancestral position of the northernmost population, especially these *T. mongolica* located in its central area. In addition, the phylogenetic position of QPC25 and HNHCQ39 was not consistent with its geographical location, which may have resulted from ILS. Besides, the collection locations of the two populations were very close to the busy roads (such as beltway and expressway), which may provide opportunities for the long-distance dispersal of seeds from other populations. Despite two groups were well supported, the phylogenetic relationships within group were recovered with low to medium support in all datasets, which may be largely attributed to the low genetic differentiation among the populations rather than the insufficient phylogenetic signals. Such a similar result was also observed in previous study, which inferred from a total of 38,097 SNPs (Cheng et al., 2020).

Although representatives of Morkillioideae and Seetzenioideae were absent, the remaining three of the five subfamilies of *Zygophyllaceae* were recovered as monophyletic with strong support. In our analyses, Larreoideae was resolved sister to *Zygophylloideae*, consistent with previous studies (Sheahan and Chase, 2000; Wu et al., 2018). To some extent, the closest phylogenetic relationship coupled with the highly similar cp genomes between *Z. xanthoxylon* and *Z. mucronatum* in our study, supported that the two species form a genus (*Zygophyllum*) rather than two genera (*Sarcozygium* and *Zygophyllum*). Such a taxonomy was also suggested in most previous studies (e.g., Beier et al., 2003; Wu et al., 2018; Zhang et al., 2021).

## Data Availability

The data presented in the study are deposited in the NCBI repository, accession number ON408217–ON408231, BioProject PRJNA866691 and PRJNA866686.

## Supplementary material

SUPPLEMENTARY FIGURE S1 IR/SC boundaries of 13 *T. mongolica* chloroplast genomes. Number above the gene features means the distance between the ends of genes and the borders sites. These features are not to scale.

SUPPLEMENTARY FIGURE S2 Synteny and rearrangements detected in six Zygophyllaceae chloroplast genomes and *T. terrestris* was set as the reference genome. Colored bars indicated syntenic blocks, connecting lines indicated the correspondence between blocks, and pink boxes indicated the IRs. The numbers indicated the genome length (bp).

SUPPLEMENTARY FIGURE S3 Visualization of the comparison of complete six Zygophyllaceae chloroplast genomes. *T. terrestris* was used as the reference genome.

SUPPLEMENTARY FIGURE S4 The ML tree of 13 *T. mongolica* inferred from the noncoding region sequences. Numbers at nodes represented the bootstrap support values and posterior probability values. Dash (–) denoted this relationship was not supported by the BI analysis.

SUPPLEMENTARY FIGURE S5 The ML tree of 13 *T. mongolica* inferred from the protein-coding region sequences. Numbers at nodes represented the bootstrap support values and posterior probability values. Dash (–) denoted this relationship was not supported by the BI analysis.

SUPPLEMENTARY FIGURE S6 The ML tree of Zygophyllaceae inferred from SSC + single IR sequences. Numbers at nodes represented the bootstrap support values and posterior probability values.

SUPPLEMENTARY FIGURE S7 The BEAST-derived chronograms of *T. mongolica* with one calibration point. The divergence time estimates based on the complete cp genome sequences. Red pentastar represented the calibration point.
