# Supplementary figures and images for: Comparative chloroplast genomics provides insights into the genealogical relationships of endangered Tetraena mongolica and the chloroplast genome evolution of related Zygophyllaceae species
Source: Front Genet. 2022 Dec 8;13:1026919. doi: 10.3389/fgene.2022.1026919 (PMC9773207; doi:10.3389/fgene.2022.1026919)

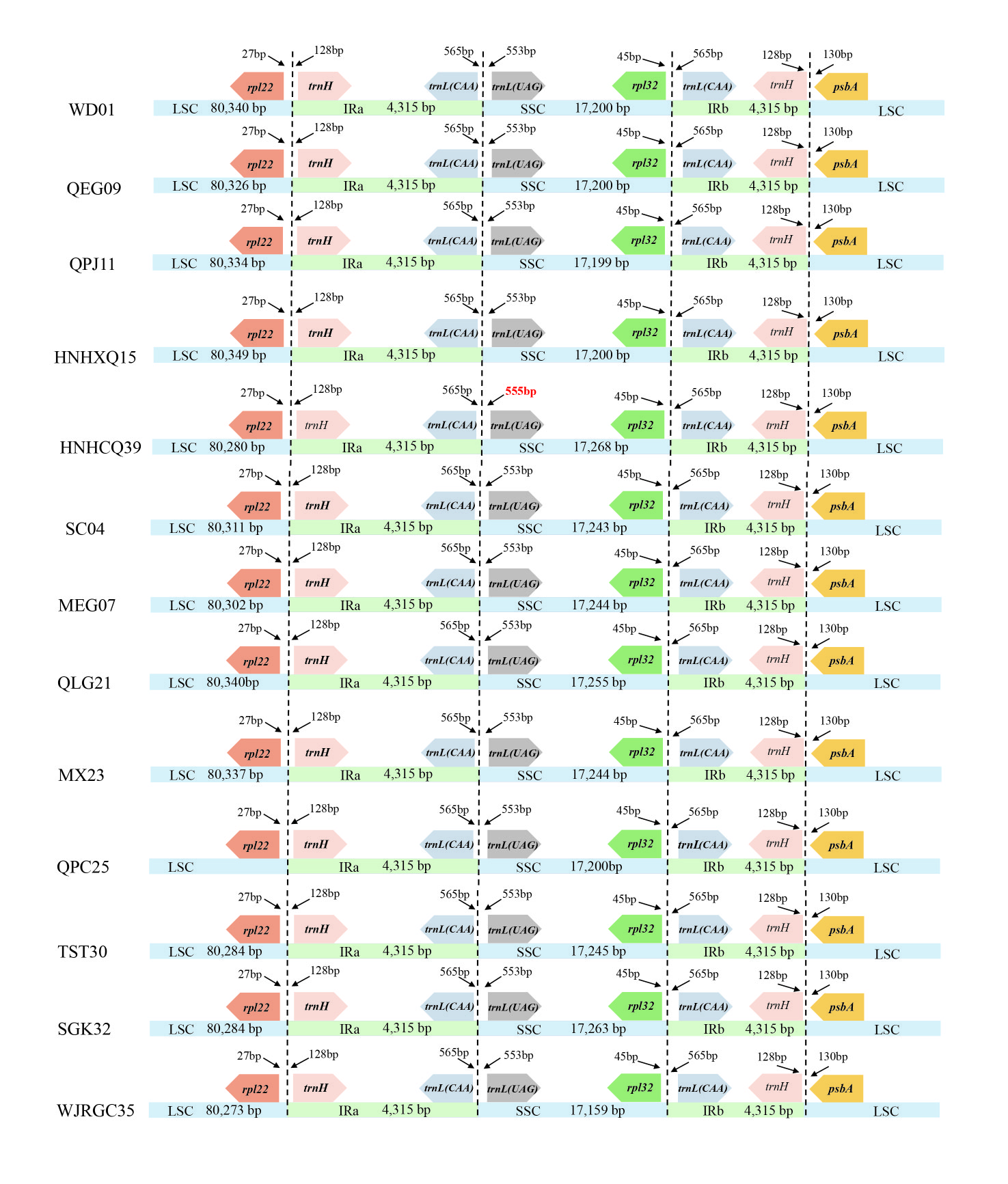

Supplement: Supplementary file 1 [file DataSheet1.zip › Supplementary material1128/image1_Figure S1.jpg]

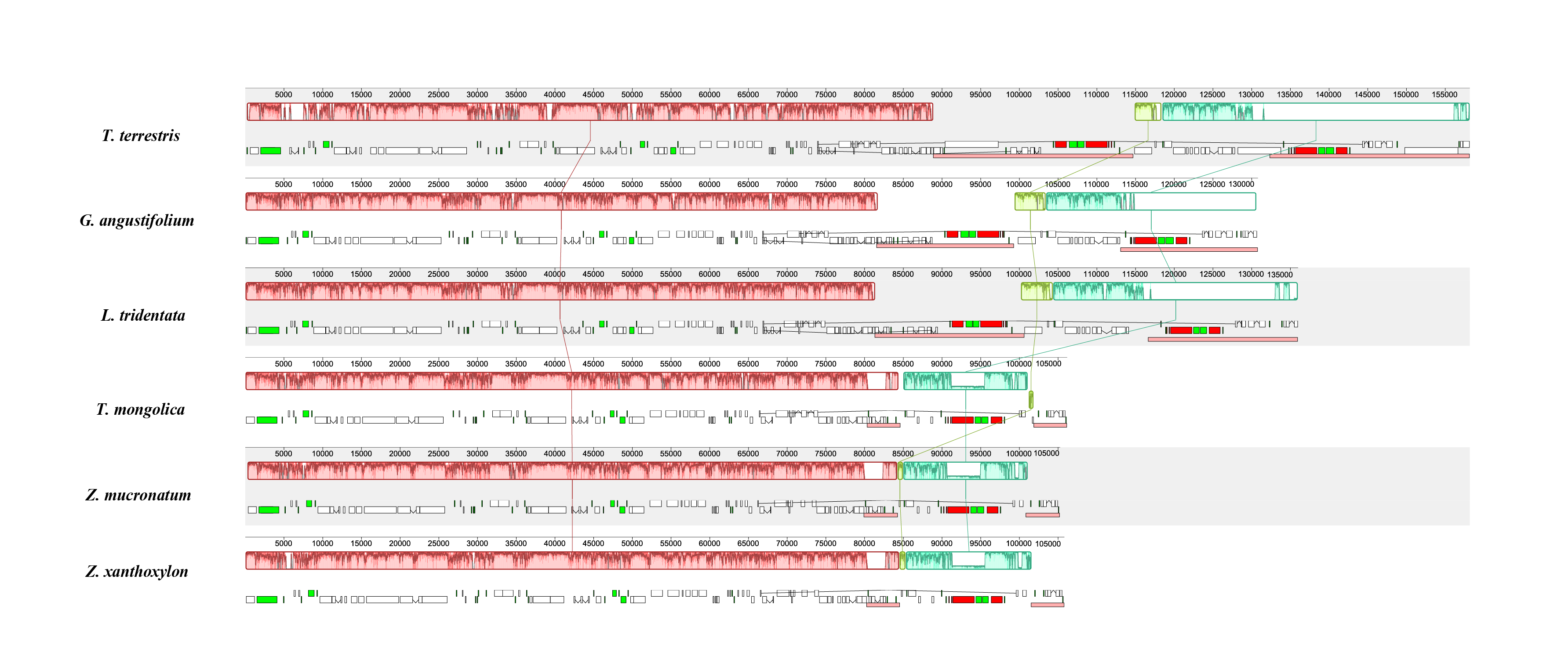

Supplement: Supplementary file 1 [file DataSheet1.zip › Supplementary material1128/image2_FigureS2.tif]

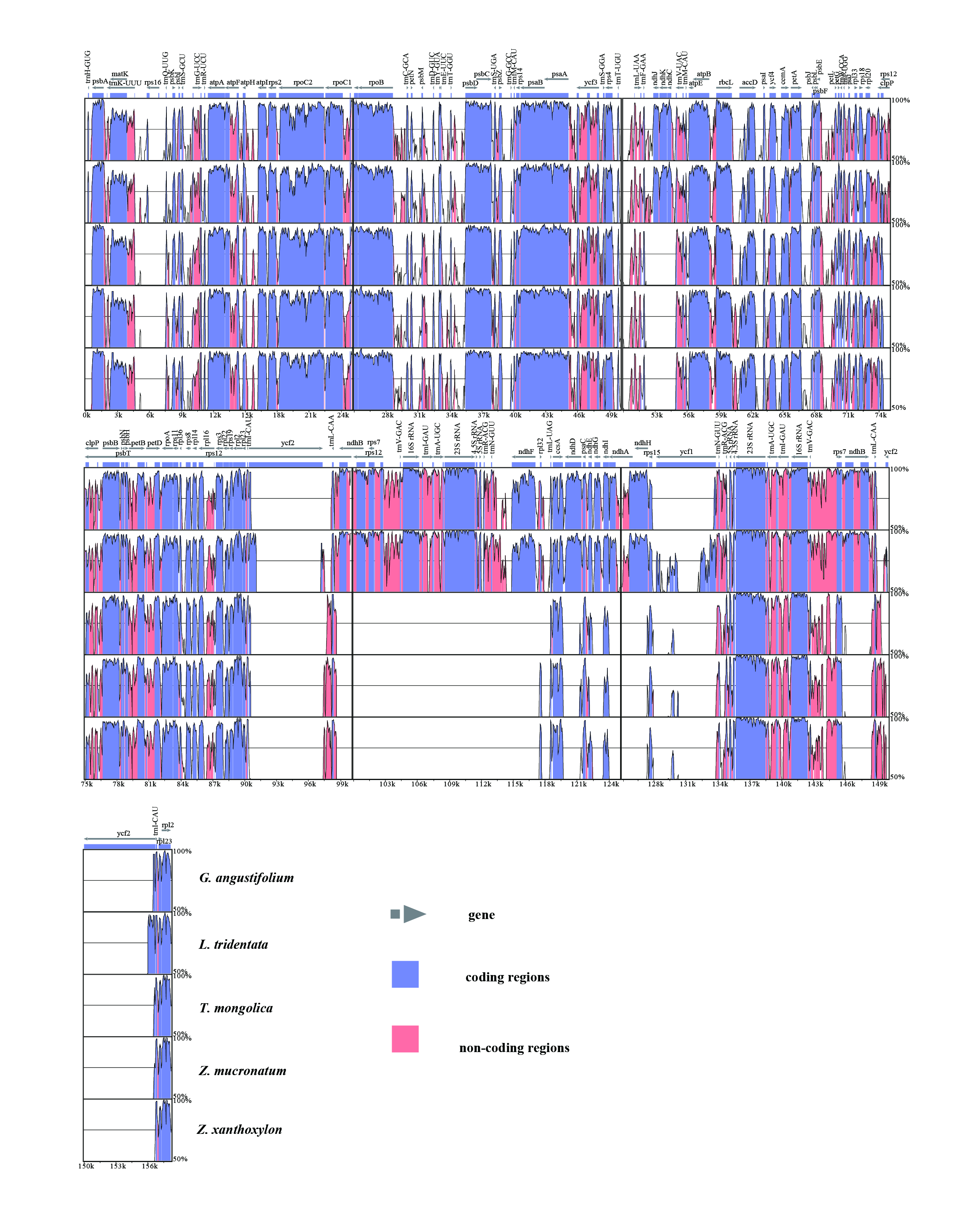

Supplement: Supplementary file 1 [file DataSheet1.zip › Supplementary material1128/image3_FigureS3.tif]

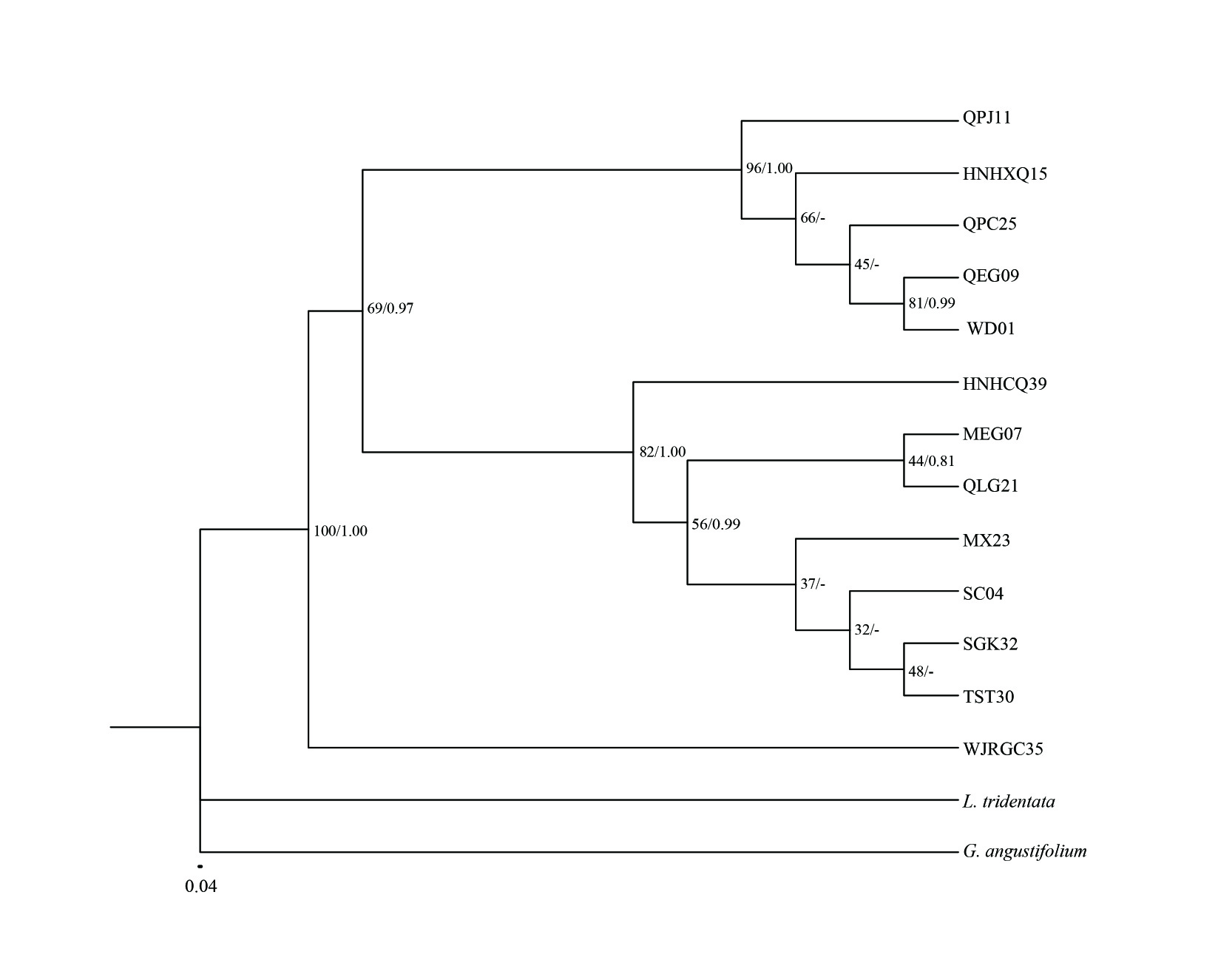

Supplement: Supplementary file 1 [file DataSheet1.zip › Supplementary material1128/image4_Figure S4.jpg]

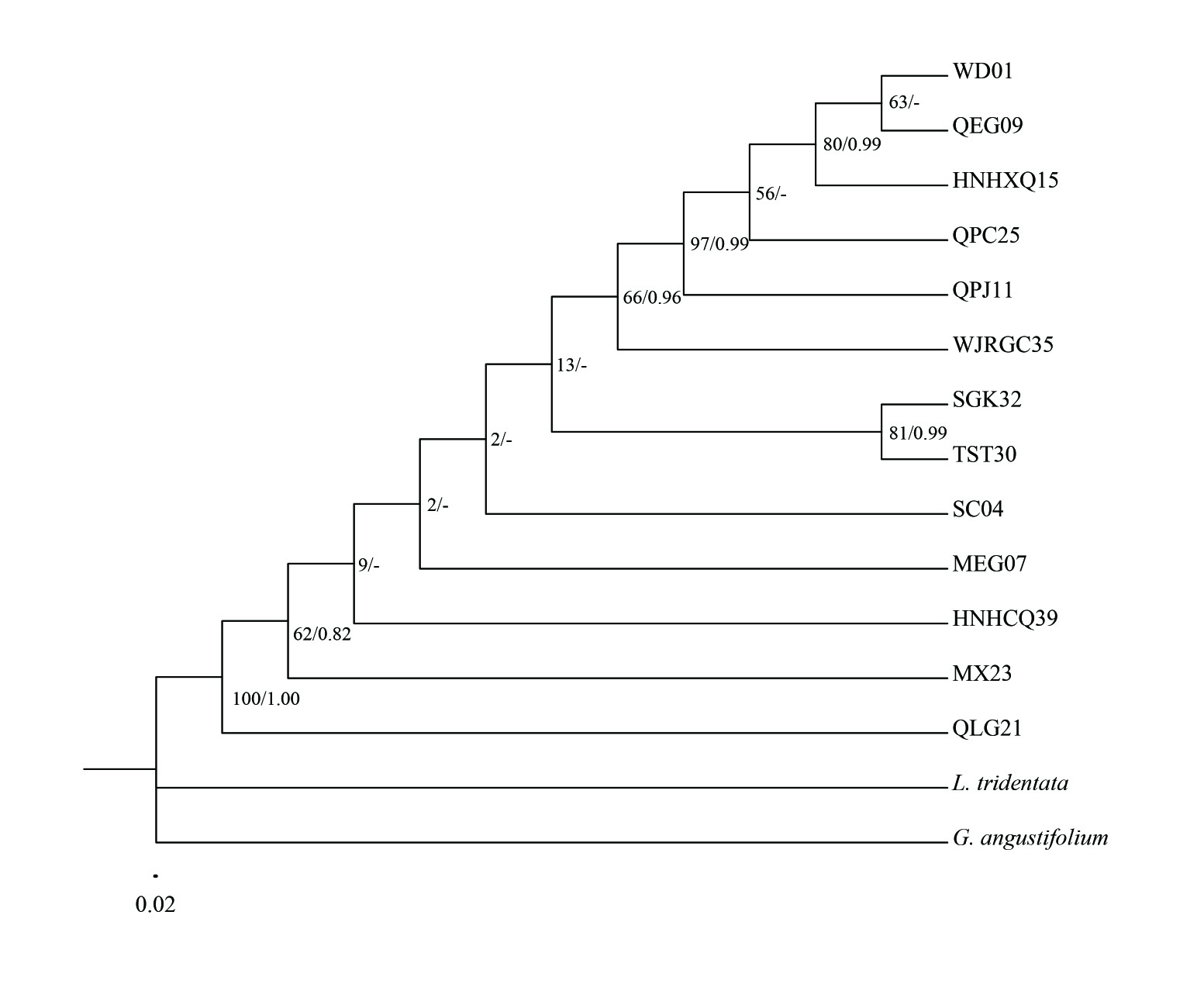

Supplement: Supplementary file 1 [file DataSheet1.zip › Supplementary material1128/image5_Figure S5.jpg]

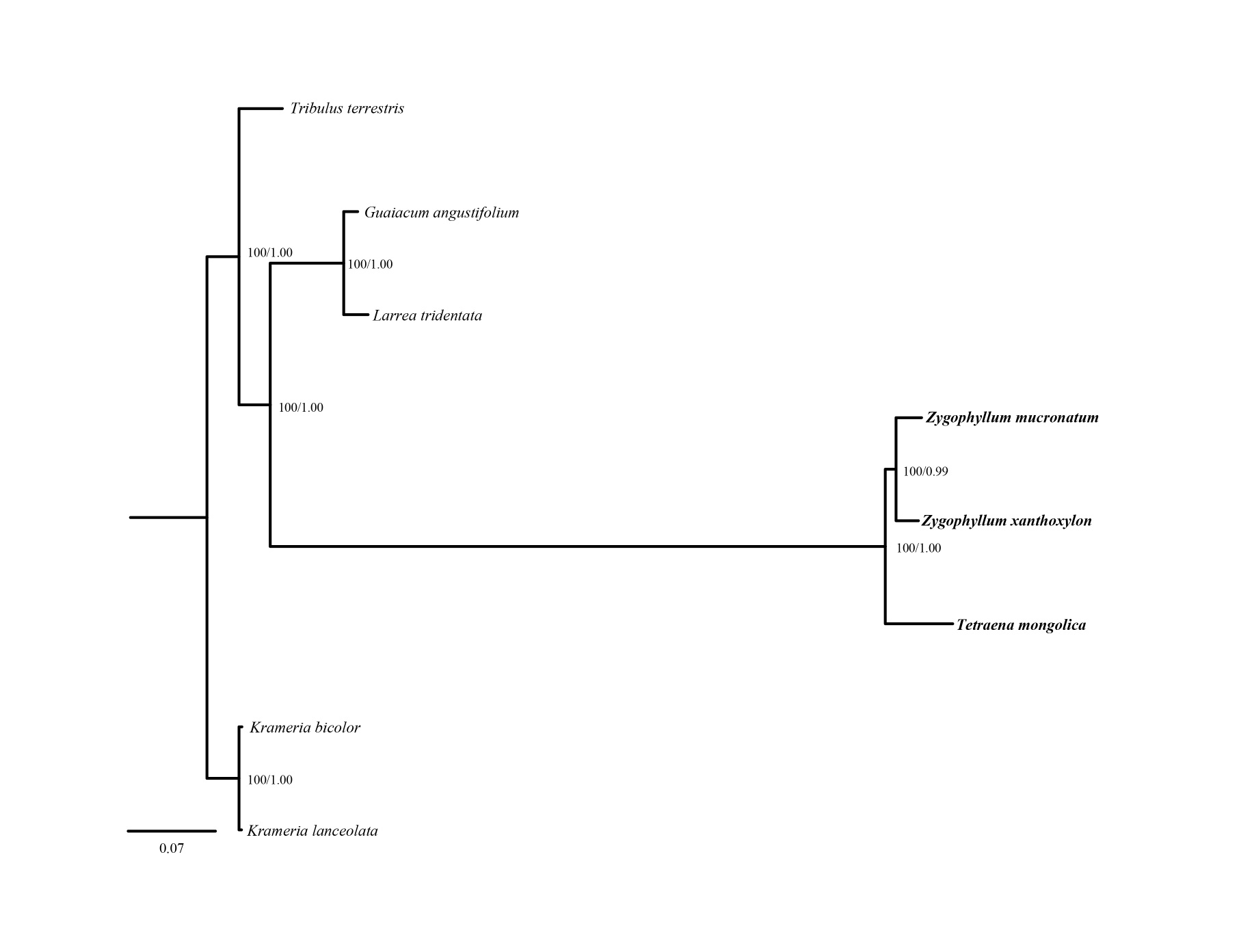

Supplement: Supplementary file 1 [file DataSheet1.zip › Supplementary material1128/image6_Figure S6.jpg]

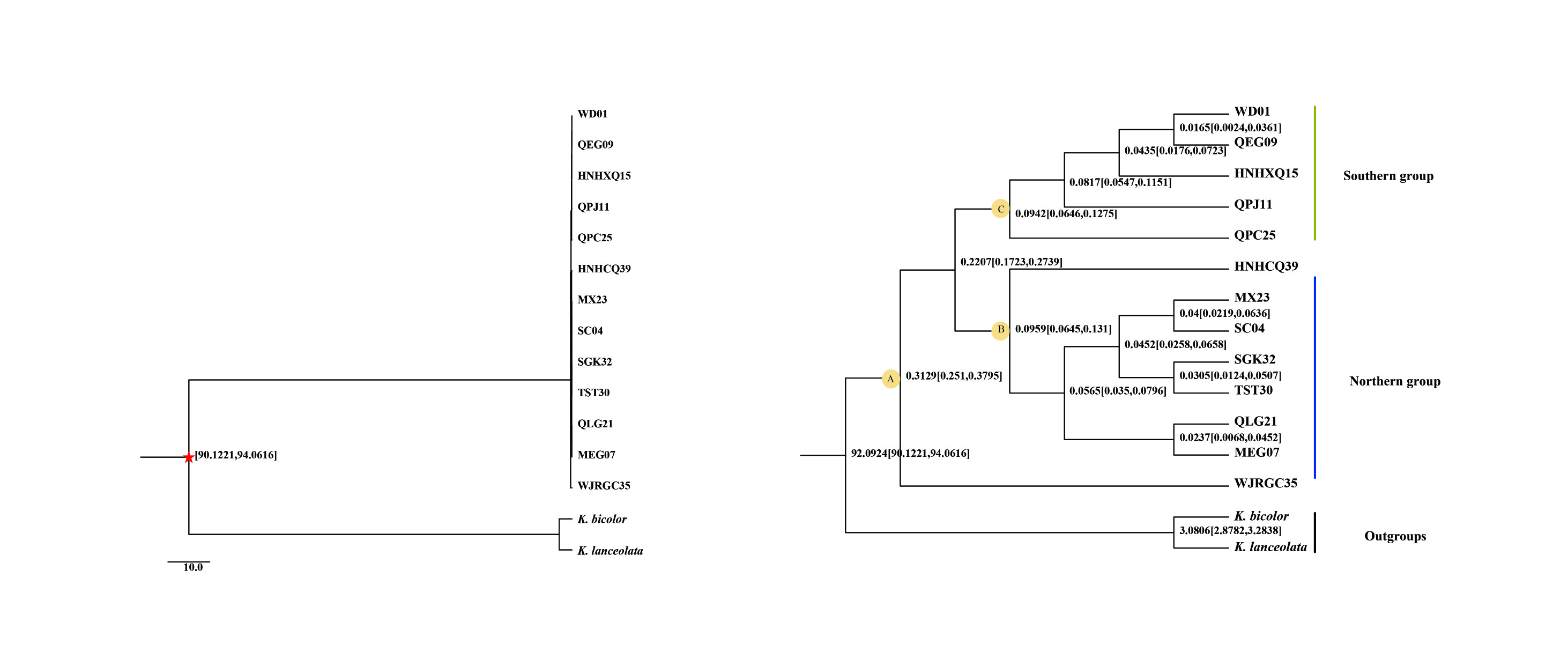

Supplement: Supplementary file 1 [file DataSheet1.zip › Supplementary material1128/image7_Figure S7.jpg]
